# Isolation and In Vitro Pharmacological Evaluation of Phytochemicals from Medicinal Plants Traditionally Used for Respiratory Infections in Limpopo Province

**DOI:** 10.3390/antibiotics14100965

**Published:** 2025-09-25

**Authors:** Potsiso L. Koma, Mashilo M. Matotoka, Ofentse Mazimba, Peter Masoko

**Affiliations:** 1 Faculty of Science and Agriculture, Department of Biochemistry, Microbiology and Biotechnology, University of Limpopo, Private Bag X1106, Sovenga 0727, South Africa; potsisoletauk@gmail.com (P.L.K.); mashilo.matotoka@ul.ac.za (M.M.M.); 2 Chemical and Forensic Sciences, Botswana International University of Science and Technology, Private Bag 16, Palapye 10017, Botswana; mazimbao@biust.ac.bw

**Keywords:** antimycobacterial, bioassay-guided isolation, phytochemicals, *Mycobacterium smegmatis*, antioxidant

## Abstract

**Background/Objectives**: Tuberculosis (TB) remains one of the most pressing global health challenges, ranking among the leading infectious causes of mortality worldwide. Medicinal plants possess antimycobacterial potential, warranting the isolation and characterization of their bioactive compounds to address bacterial infections. The study aimed to determine five selected traditional medicinal plants’ in vitro antioxidant and antibacterial activities and the isolation of active phytoconstituents. **Methods**: Powdered leaf material was extracted using *n*-hexane, dichloromethane, acetone, methanol, and water. The quantity of phytochemicals and antioxidants was determined using colorimetric assay, The antimycobacterial activity and combination effects were determined using microbroth dilution assay. Cell viability was determined using the [3-(4,5-dimethylthiazol-2-yl)-2,5-diphenyltetrazolium bromide] MTT reduction assay. Bioassay-guided fractionation was used to isolate bioactive compounds. **Results**: Polar solvents had high extraction yields, and all extracts had varying phytoconstituents. Active extracts were selected for fractionation and isolation of pure compounds using gradient elution column chromatography. *Rhoicissus tridentata* water extracts had the highest total phenolic (335.20 ± 8.26 mg GAE/g) and tannin (103.48 ± 7.36 mg GAE/g) content, while *Rosmarinus officinalis* (45.90 ± 11.04 mg QE/g) methanol extract had the highest total flavonoid. *Ximenia caffra* had promising antioxidant activity. *R. officinalis* had prominent antimycobacterial. *Rhoicissus tridentata* had the highest percentage cell viability. Two compounds were isolated, and they were active against *Mycobacterium smegmatis* with minimum inhibitory concentration values ranging from 0.125 to 0.25 mg/mL. **Conclusions**: The selected medicinal plants contain phytochemicals with antioxidant and antimycobacterial activities, supporting their pharmacokinetic studies and evaluation against *Mycobacterium tuberculosis* H37Rv.

## 1. Introduction

Tuberculosis (TB) is an airborne infectious bacterial disease caused by *Mycobacterium tuberculosis*, which primarily affects the lungs [1]. In 2021, 10.6 million people were infected with TB, and 1.6 million deaths were reported globally [2]. The World Health Organization (WHO) has raised concerns about microbial infections that are resistant to currently available treatment methods, primarily due to the inappropriate, irregular, and excessive use of antibiotics [3]. Additionally, microbial resistance arises from continuous selective pressure and the ability of bacteria to develop new survival strategies against newly developed antibiotics [4].

*M. tuberculosis* is classified as extensively drug-resistant (XDR) and multidrug-resistant (MDR) due to its resistance to both first- and second-line TB medications [5]. Isoniazid, rifampicin, ethambutol, and pyrazinamide are used in TB treatment, typically administered for two months, followed by isoniazid and rifampicin for an additional four months [6]. Despite the availability of these first-line antibiotics, tuberculosis remains one of the leading causes of infectious disease mortality worldwide. Moreover, these drugs are associated with various side effects, which often hinder patient adherence to treatment [7], with approximately 8–85% of TB patients experiencing side effects ranging from mild to severe [8]. This growing challenge highlights the urgent need for new, safer, and more effective anti-TB agents.

The environmentally benign *Mycobacterium smegmatis* is a commonly used model organism for studying the metabolism and pathogenicity of mycobacteria [9]. Due to its close genetic and biochemical relationship with *M. tuberculosis*, it is frequently used in TB research [10]. These species share orthologous genes; approximately 96% of the genes essential in *M. smegmatis* are also present in *M. tuberculosis*, with 90% of them being essential in the latter. Their genomic architecture is highly conserved, and similar gene co-localization patterns suggest that gene regulatory mechanisms have been preserved across both species [11].

Macrophages infected with *M. tuberculosis* increase the production of reactive oxygen species (ROS) as a defense mechanism to destroy the bacteria. Under physiological conditions, elevated ROS levels are typically counteracted by increased antioxidant synthesis to mitigate tissue damage [12]. Antioxidant and antimicrobial activities can be enhanced by evaluating the synergistic effects of combined agents, thereby improving overall therapeutic efficacy [13].

Medicinal plants have been used for thousands of years in health maintenance and remain a primary source of healthcare [14]. In antibiotic drug discovery, they have been instrumental, with 80% of all synthetic drugs derived from plant-based sources [15]. Compounds from medicinal plants such as tannins, terpenoids, phenols, steroids, flavonoids, and aromatic compounds are frequently used in the food, pharmaceutical, and cosmetic industries. These chemical substances, known as secondary metabolites, play a vital role in plant growth and development, acting as defense compounds that aid in environmental adaptation [16]. Phytochemicals possess a wide range of pharmacological benefits and have demonstrated promising biological activities, including wound healing, antidiabetic, antioxidant, cell migration/proliferation, and antimicrobial effects [17].

Bioactive compounds from medicinal plants can be isolated and purified using column chromatographic methods. Traditionally, the analysis of compound fractions via column chromatography is supported by thin-layer chromatography (TLC). Various analytical instruments, such as silica gel column chromatography and TLC, are commonly used for separating bioactive molecules [18]. The purified compounds can then be identified using a range of spectroscopic techniques, including mass spectrometry, nuclear magnetic resonance (NMR) spectroscopy, infrared (IR) spectroscopy, and UV–visible spectroscopy [19].

Several indigenous plants are traditionally used in Southern Africa for the management of TB and related respiratory conditions. *Rhoicissus tridentata* (L.f.) Wild & Drumm. subsp. *cuneifolia* (Eckl. & Zehr.), N.R. Urton is a well-documented example. Species in this genus produce diverse phytochemicals such as alkaloids, terpenoids, and polyphenols, which have been linked to antioxidant, anti-inflammatory, and anticancer properties [20]. The plant typically grows along forest boundaries and in open grasslands, reaching up to two meters in height, and preparations from its tubers, leaves, and stems are widely used to relieve TB symptoms [21,22]. *Zanthoxylum capense* Thunb. (Rutaceae family) is a shrub or small tree, easily recognized by its sharp thorns and citrus-scented foliage and fruits [23]. It is distributed mainly in the eastern regions of Southern Africa, where its fruits and leaves are used for treating cough and other minor ailments [24]. *Ziziphus mucronata* Willd. (Rhamnaceae), occurs across Africa, Asia, Australia, and the Americas [25]. Its bark, leaves, and roots are commonly used in folk medicine for managing TB-associated infections, rheumatism, gastrointestinal disorders, and snake bites. Root infusions are also administered for gonorrhea, diarrhea, and dysentery [26]. Preparations from the bark are further employed for pain relief and the treatment of cough, respiratory tract infections, and chest problems [22], with additional evidence highlighting its role in managing chest complications [27]. *Ximenia caffra* Sond. (Ximeniaceae; previously Olacaceae), also known as the sour plum, is represented by two main varieties: *X. caffra* var. *caffra*, which retains hairy leaves until maturity, and *X. caffra* var. *natalensis*, with smooth and hairless leaves [28]. The species is a widely distributed, indigenous, and native to Central, Southern, and Eastern Africa, including Madagascar [29]. Its hairless variety bears bluish-green oblong leaves that fold upwards and produces clustered greenish-white flowers on the axils of spines [30]. Traditional applications include the use of its leaves and roots for treating constipation, stomach pain, and leprosy [31]. Finally, *Rosmarinus officinalis* L. (rosemary, Lamiaceae) is an aromatic herb native to the Mediterranean region. Beyond its culinary importance, it has significant medicinal value as an antioxidant, hepatoprotective agent, and in the management of Alzheimer’s disease and angiogenesis [32]. In ethnomedicine, decoctions prepared by briefly boiling the plant are taken orally for ailments such as chest pain, cough, and fever [33].

The aim of this study was to evaluate the in vitro antioxidant and antibacterial properties of five medicinal plants traditionally used in the Limpopo Province of South Africa for managing respiratory infections, including tuberculosis. The focus was to identify and quantify key phytochemicals within these plants, assess their bioactivity against *M. smegmatis* as a surrogate for *M. tuberculosis*, and isolate active phytoconstituents using bioassay-guided fractionation. By integrating phytochemical screening, antimicrobial assays, and compound isolation techniques, the research seeks to provide scientific validation for traditional medicinal practices and to identify potential lead compounds for further pharmacological development.

## 2. Results

### 2.1. Extraction

**Figure 1:**
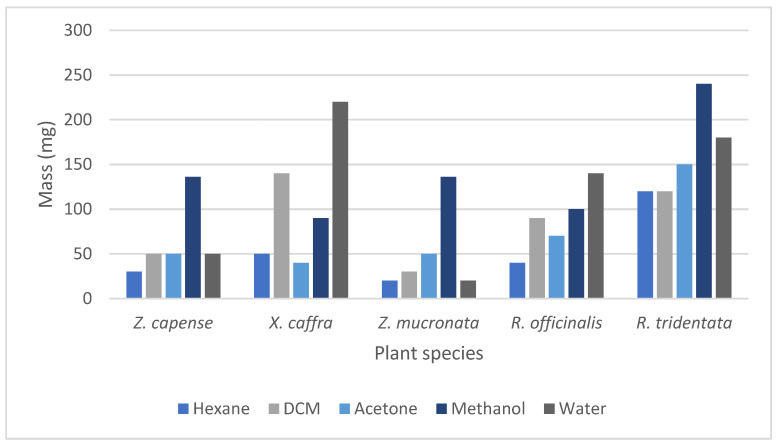
The mass of the *n*-hexane, dichloromethane (DCM), acetone, methanol, and water extracts from 1 g of powdered leaves of the selected plants.

Given the traditional use of *R. tridentata*, *R. officinalis* L., *X. caffra*, *Z. capense*, and *Z. mucronata* (Table 1), the leaves were selected for investigation. Extraction results indicated that methanol was the most efficient solvent, yielding the highest amount of extract for *Z. capense* (136 mg), *Z. mucronata* (136 mg), and *R. tridentata* (240 mg). In contrast, water showed to be the most effective for *R. officinalis* (140 mg) and *X. caffra* (220 mg) (Figure 1).

### 2.2. Quantification of Phytoconstituents

*R. tridentata* water extracts had the highest amounts of both total phenolic content (335.20 ± 8.26 mg GAE/g) and tannin content (103.48 ± 7.36 mg GAE/g) (Table 2). In contrast, *R. officinalis* methanol crude extract had the highest total flavonoid content (45.90 ± 11.04 mg QE/g).

### 2.3. Antioxidant Activity

The *n*-hexane and dichloromethane extracts of *X. caffra*, the *n*-hexane and methanol extracts of *R. officinalis*, and the *n*-hexane extract of *R. tridentata* exhibited EC_50_ values comparable to that of ascorbic acid (0.0038 µg/mL) (Table 3).

### 2.4. Antimycobacterial Activity

The hexane and dichloromethane extracts of *R. tridentata* had MICs of 0.16 and 0.31 mg/mL, respectively. The acetone and methanol extracts of *Z. mucronata* had MICs of 1.25 mg/mL. *X. caffra* hexane extract was active against *M. smegmatis* with an MIC value of 0.31 mg/mL. *Z. capense* was not active against *M. smegmatis* (Table 4).

### 2.5. Combinational Effects of Extracts

The combination of *R. officinalis* hexane extract with the hexane extract of either *Z. mucronata*, *Z. capense*, *X. caffra*, or *R. tridentata* exhibited prominent activity, with a minimum inhibitory concentration (MIC) value of 0.63 mg/mL (Table 5). All combinations showed antagonistic interactions except for the combination of *Z. mucronata* and *Z. capense*, which displayed an indifferent outcome.

### 2.6. Cytotoxicity of Plant Extracts

**Figure 2:**
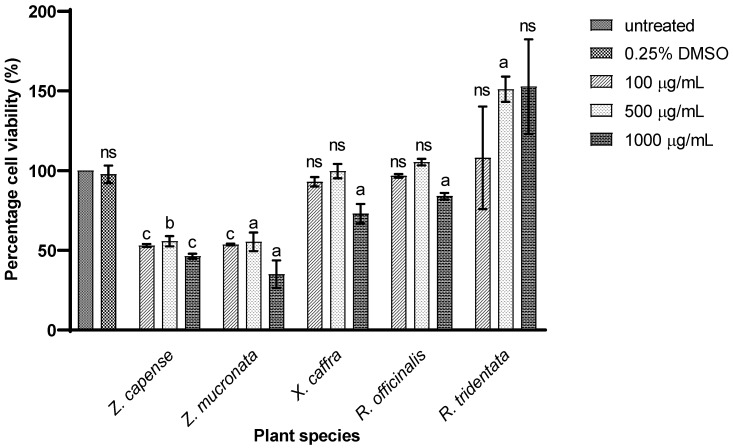
The cytotoxic effects of plant acetone extracts of *Z. capense*, *Z. mucronata*, *X. caffra*, *R. officinalis*, and *R. tridentata* on THP-1 cells. Two-way ANOVA was used to analyze the data, and then the Tukey multiple comparison post hoc test was performed. ns = non-significant, (a, b, and c) = level of significance compared to untreated cells.

No significant difference in cell viability after incubation when comparing untreated cells to those treated with *X. caffra* (at 100 and 500 µg/mL), *R. tridentata* (at 100 and 1000 µg/mL), and *R. officinalis* (at 100 and 500 µg/mL). However, a notable reduction in cell viability was observed for cells exposed to *Z. capense* and *Z. mucronata* at all tested concentrations, as well as *R. tridentata* (at 500 µg/mL), *X. caffra* (at 1000 µg/mL), and *R. officinalis* (at 1000 µg/mL) (Figure 2).

### 2.7. Isolation of Bioactive Compounds from R. officinalis Leaves

The hexane extracts were the most potent, with H2 and H3 showing MIC of 0.156 mg/mL. The H1 extract was also effective, with an MIC of 0.182 mg/mL. The dichloromethane extracts were less active (MIC, 0.3125 mg/mL), while the first acetone extract (A1) had a lower potency (MIC, 1.125 mg/mL). The methanol extracts were found to have the least activity among all the extracts tested (Table 6). Among the isolated compounds, Compound **1** was the most effective, demonstrating an MIC of 0.125 mg/mL. This value was two-fold lower than the MIC of the other isolated compound (Table 6).

### 2.8. Structural Elucidation of Isolated Compounds

**Figure 3:**
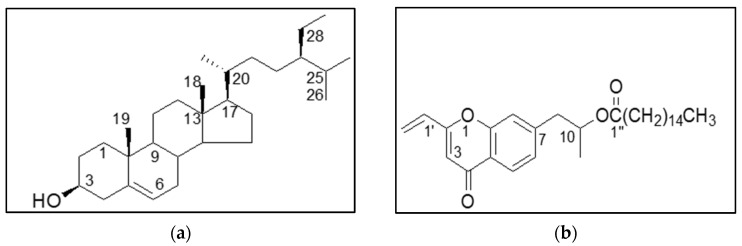
Structures of the isolated compounds: (**a**) β-Sitosterol; (**b**) 1-(4-oxo-2-vinyl-4H-chromen-7-yl)propan-2-yl palmitate.

Compound **1** showed the common plant metabolite β-sitosterol (Figure 3a). Similarly, the NMR data of Compound **2** was identified as a chromone derivative (Figure 3b).

## 3. Discussion

Efficient extraction of bioactive compounds is critical for maximizing the biological activity of plant extracts [35]. In this study, the results align with previous findings, indicating that polar solvents often result in higher extraction yields due to their ability to solubilize a wide range of phytochemicals, especially polyphenols [36]. The high yields in methanol and water suggest that the targeted plant species predominantly contain polar constituents. Ultimately, the choice of solvent influenced not only the total extract yield but also the potential biological activity of the extracts, highlighting the importance of solvent selection in bioassay-guided phytochemical studies [37].

Bioactive compounds including flavonoids, phenols, anthocyanins, ascorbic acid, amides, alkaloids, tannins, saponins, and glycosides from different parts of medicinal plants play an important role in human health due to their biological activities [38]. The solubility of phenolic compounds is higher in polar solvents such as water and ethanol, or their combination [39]. Naidoo et al. [40] reported that *R. tridentata* contains polyphenolic compounds. A study by Kudamba et al. [41] found that *R. tridentata* had higher polyphenolic and tannin content in methanol extracts compared to aqueous extracts, which contradicts the findings of this study. This discrepancy may be attributed to differences in environmental and geographical conditions, as their study involved samples collected from different countries. Environmental variables such as climate, soil composition, altitude, and ecological stress have been shown to significantly influence the phytochemical profile of plants [16]. Similarly, our findings for *R. officinalis* are consistent with Al-Jaafreh et al. [42], who reported high flavonoid levels in ethanol and methanol extracts. The total flavonoid content for the dichloromethane extract of *Z. capense* was higher than its total phenolic content. Bui et al. [43] reported similar results for the dichloromethane extract of *Avicennia officinalis*, which had higher flavonoid content (61.04 ± 0.02 mg QE/g) than phenolic content (43.05 ± 0.01 mg GAE/g). Dichloromethane is generally more effective at extracting non-polar compounds [44]. Flavonoids are a key class of plant polyphenols known for their potent antioxidant activity and metal-chelating properties. These bioactivities contribute to their therapeutic potential in managing oxidative stress-related conditions and inflammation during infections [45].

*M. tuberculosis* is an intracellular organism that causes oxidative stress, leading to cell degradation, mutations, tissue damage, and an immunocompromised state [46]. The delicate balance between oxidants and antioxidants is essential for maintaining tissue homeostasis. Although initial *Mycobacterium tuberculosis* infection results in high levels of ROS production, there are cases where antioxidant levels do not rise proportionately, leading to an imbalance between ROS and antioxidants in tissues [12]. Oxidative stress is defined as an imbalance that favors oxidants over antioxidants. Reducing these oxidants is considered an effective strategy for lowering oxidative stress levels in organisms [47]. Antioxidants are substances that can delay or stop the oxidation of a substrate when present in smaller amounts than the oxidizable substrate. Their actions include reducing malignant transformations, DNA mutations, oxidative stress, and other indicators of cell damage [48].

The plant extracts’ ability to reduce free radicals was evaluated using the DPPH and FRAP assays. Antioxidant potential was represented by EC_50_ values, indicating the concentrations at which the extracts exhibit half-maximal inhibitory activity [49]. The *X. caffra* (hexane and dichloromethane), *R. officinalis* (hexane and methanol), and *R. tridentata* (hexane) extracts had EC_50_ values comparable to that of ascorbic acid (0.0038 µg/mL) (Table 3). This indicates that non-polar extracts had higher radical scavenging activity compared to polar extracts. The lowest EC_50_ value was for ascorbic acid, followed by the dichloromethane extract of *X. caffra* (0.0039 µg/mL).

The ferric reducing antioxidant power (FRAP) assay is important for screening and determining the efficacy of antioxidant compounds. It is based on the reduction in a colorless Fe^3+^ complex into an intense blue Fe^2+^ once it interacts with a potential antioxidant [50]. Results were recorded as EC_50_ values. *X. caffra* extracts exhibited relatively high reducing power across all solvent types compared to ascorbic acid (3.042 µg/mL) and other plant extracts. Jacob et al. [51] reported the potential antioxidant activity of *X. caffra* from its polar methanolic extract. Polyphenolic compounds (phenolics and flavonoids) concentrated in the extracts were responsible for this antioxidant activity [52]. Nyila [53] demonstrated that *X. caffra* exhibited the highest ABTS antioxidant activity with an IC_50_ value of 6.5 μg/mL. Essential oil from *R. officinalis* has been reported to exhibit strong radical scavenging activity, with an IC_50_ value of 77.6 μL/mL [54]. Ouknin et al. [55] found that *R. officinalis* had antioxidant activity with IC_50_ values of 337.23 ± 3.50 and 265.45 ± 1.36 µg/mL in DPPH and FRAP assays, respectively. *Rhoicissus tridentata* subsp. *cuneifolia* has also demonstrated antioxidant activity, with a DPPH IC_50_ of 0.06 μg/mL [56].

The lower the MIC value of an extract, the greater its antimicrobial activity. Plant extracts with significant antibacterial activity typically show MIC values <1 mg/mL [57]. MIC values of 0.08 mg/mL were obtained from *R. officinalis* (hexane, dichloromethane, acetone, and methanol) extracts excluding the water extract, implying high activity against *M. smegmatis* (Table 4). Dheyab et al. [58] previously demonstrated that *R. officinalis* was active against *M. tuberculosis*, while the water extract had a very high MIC value. Essential oils of *R. officinalis* have also shown antibacterial activity against *S. aureus*, *E. coli*, and *K. pneumoniae* [59]. Its traditional use in treating cough and flu, as reported by Borges et al. [60], may explain its antimycobacterial activity. Hexane and dichloromethane extracts of *R. tridentata* had MICs of 0.16 and 0.31 mg/mL, respectively. This differs from reports where methanolic extracts of *R. tridentata* exhibited MICs of 0.13 mg/mL against *S. marcescens* [57]. The hexane extract of *X. caffra* was also active against *M. smegmatis*, with an MIC value of 0.31 mg/mL. However, *Z. capense* showed no activity against *M. smegmatis*. Bacterial growth inhibition is attributed to the presence of bioactive compounds in the plant extracts [61]. The acetone and methanol extracts of *Z. mucronata* showed MICs of 1.25 mg/mL, differing from results by Wa Ilunga et al. [62], where no activity against *M. smegmatis* was reported. The chemical profile of herbal materials can be influenced by factors such as genetic composition, developmental stage, and environmental conditions [63].

When multiple antimicrobials are combined, they may exhibit synergistic, antagonistic, or indifferent effects compared to their individual actions [64]. Since the hexane extracts showed good antibacterial activity, they were selected for combination testing in a 1:1 ratio. Combining *R. officinalis* hexane extract with the hexane extract of *Z. mucronata*, *Z. capense*, *X. caffra*, or *R. tridentata* yielded prominent activity, with MIC values of 0.63 mg/mL (Table 5). All combinations exhibited antagonistic outcomes, except for the combination of *Z. mucronata* and *Z. capense*, which was indifferent. Antagonistic effects occur when combined agents exhibit lower activity than expected, often due to interference in mechanisms of action, competition for cellular targets, chemical incompatibility, or opposing biological activities, warranting further investigation [65,66]. These findings are consistent with previous studies, where *Z. mucronata* ethanol extract combined with chloramphenicol or tetracycline showed indifferent effects against *K. pneumoniae*, *A. calcoaceticus*, and *P. aeruginosa* [26]. This underscores that not all plant extract combinations enhance efficacy, emphasizing the need to evaluate interactions before proposing anti-TB therapeutic combinations.

Medicinal plants may also contain toxic bioactive compounds [67]. Cytotoxicity testing is essential to assess safety by detecting loss of membrane integrity related to cell death. Toxicity can vary depending on dosage, growth stage, administration method, solubility, and frequency of exposure [30]. In this study, THP-1 cells were chosen and differentiated using PMA. Upon incubation with PMA, THP-1 monocytes differentiate into macrophages [68].

To investigate *M. tuberculosis*–macrophage interactions, several in vitro systems have been established. THP-1 monocytes are widely used as a model for polarized monocyte-derived macrophages [69]. The solvent control (0.25% DMSO) did not significantly reduce cell viability compared to untreated cells (Figure 2), indicating that observed cytotoxic effects were due to plant extracts, not the solvent. Establishing solvent safety is critical for accurately interpreting TB drug discovery assays. Among the tested extracts, the acetone extract of *R. tridentata* showed the highest cell viability, whereas *Z. mucronata* and *Z. capense* demonstrated toxicity at 1000 µg/mL, with viability dropping below 50%. Isolated stigmasterol from *R. tridentata* has been reported as non-toxic to breast cancer cells, although its synthetic analogs showed enhanced toxicity [20]. Mansour et al. [70] reported no toxicity in THP-1 cells when treated with *R. officinalis* at concentrations below 608.7, 1356, and 3296 µg/mL. Similarly, Rathore et al. [71] demonstrated no cytotoxic effects of *R. officinalis* essential oils against A549 and CAL 27 cancer cell lines.

Serial exhaustive extraction facilitates the recovery of a broad range of bioactive compounds while preserving their chemical and biological properties [72]. In this study, ground *R. officinalis* leaves (1 kg) were subjected to serial exhaustive extraction using solvents of increasing polarity: n-hexane, dichloromethane, acetone, and methanol. Methanol produced the highest yield (71.63 g), followed by dichloromethane (63.71 g), *n*-hexane (50.13 g), and acetone (30.94 g). The MIC values of the extracts against *M. smegmatis* were determined using a serial broth microdilution assay. Among the extracts, the hexane fractions (H2 and H3: 0.156 mg/mL; H1: 0.182 mg/mL) exhibited the highest potency, followed by the dichloromethane extract (0.3125 mg/mL) and the first acetone extract (A1: 1.125 mg/mL). Methanol extracts showed the weakest antibacterial activity (Table 5).

Bioassay-guided fractionation of plant extracts, combined with chromatographic techniques, facilitates the isolation of biologically active compounds [73]. Multiple mobile phases of increasing polarity enable more effective separations. Thin-layer chromatography (TLC) has long been employed to analyze fractions from column chromatography, and silica gel chromatography remains a staple for separating bioactive molecules [74]. Although fractions 4 and 5 showed notable antimycobacterial activity, they were not selected for further purification due to their complexity. Instead, fractions obtained between 10% *n*-hexane:90% ethyl acetate and 70% ethyl acetate:30% methanol (total yield: 17.46 g) were selected for a second round of open-column chromatography. These fractions were prioritized based on bioautographic analysis, which revealed identical active constituents and similar chemical profiles. The 80% *n*-hexane:20% ethyl acetate solvent system provided optimal separation. The second column was packed with silica gel, and 600 subfractions were collected in 8 mL test tubes.

Subfractions with similar chemical profiles were pooled into four groups: Group 1 (tubes 1–120), Group 2 (121–240), Group 3 (241–360), and Group 4 (361–600). TLC confirmed antimycobacterial activity in all pooled groups. Groups 3 and 4 were combined due to shared chemical features and subjected to preparative TLC to isolate bioactive metabolites. The combined crude extract was applied to TLC plates, developed in 80% *n*-hexane in ethyl acetate, and visualized under UV light at 254 and 365 nm. Visible bands were marked, scraped off, and extracted from silica using ethyl acetate, followed by vortexing and filtration through cotton wool. Antibacterial activity of the purified compounds was determined using broth microdilution assays. Rifampicin served as the positive control, while acetone was the negative control. Among the isolates, Compound **1** displayed the strongest effect with an MIC of 0.125 mg/mL, which was two-fold lower than the other isolated compound (Table 6).

β-Sitosterol, isolated from *Odontonema strictum*, has been reported to inhibit *S. aureus* at 2.208 mg/mL [75]. It has also been isolated from *Malva parviflora* root bark and shown to have antibacterial properties [76]. Nandi et al. [77] reported β-sitosterol to be a safe, efficient, and non-toxic compound with substantial pharmacological potential. It is considered a valuable nutritional supplement with therapeutic applications against various communicable and non-communicable diseases [78]. Compound **2** was identified as a chromone derivative: 1-(4-oxo-2-vinyl-4H-chromen-7-yl)propan-2-yl palmitate. Chromones have been isolated from different plants such as *Aquilaria sinensis* and *Aquilaria filaria* which inhibit NO production [79]. As oxygen-containing heterocycles with a benzo-γ-pyrone structure, chromones exhibit antitumor, antimicrobial, and antiviral activities [80].

Major bioactive constituents in rosemary (*R. officinalis*) leaves such as rosmarinic acid, carnosic acid, and carnosol contribute to antioxidant, anti-inflammatory, and anticarcinogenic effects [81]. Other phytochemicals include camphor, caffeic acid, ursolic acid, betulinic acid [82], 4′,7-dimethoxy-5-hydroxy-flavone, 12-methoxy-trans-carnosic acid, and 12-methoxy-cis-carnosic acid [83]. This is the first study to report the isolation of 1-(4-oxo-2-vinyl-4H-chromen-7-yl)propan-2-yl palmitate from *R. officinalis* leaves. Nweze et al. [84] reported that sitosterol isolated from *Punica granatum* exhibited activity against *E. coli* (MIC: 6.25 μg/mL) and *Salmonella typhi* (MIC: 12.5 μg/mL). Fernando et al. [85] found β-sitosterol to have an MIC of 512 ppm against *S. aureus* and *E. coli*.

Compound **2** was confirmed to be a chromone derivative by comparison with previously published data [84,86,87,88]. The ^1^H-NMR spectrum showed ABX coupling system signals at δ 7.12 (dd, J = 2.5, 8.6 Hz), 7.33 (t, J = 2.0 Hz), and 7.74 (d, J = 8.4 Hz), indicating a chromone ring. This chromone featured a long chain and vinyl group. Vinylic protons were observed at δ 5.77 (m) and δ 4.91 and 4.99 (dd), corresponding to methylene protons. HMBC correlations between H-2′ (δ 4.91) and C-2 confirmed the vinyl linkage. The long chain was established as a palmitic acid derivative previously identified from *R. officinalis* [89], attached to C-10 via HMBC correlation between H-11 (δ 1.23, m) and C-1″ (δ 173.0). Based on NMR and calculated values, compound **2** was identified as 1-(4-oxo-2-vinyl-4H-chromen-7-yl)propan-2-yl palmitate (C_30_H_44_O_4_) (Figure 3b).

The NMR data for Compound **1** aligned with literature reports [90], supporting its identification as β-sitosterol (Figure 3a). β-Sitosterol has been reported to exhibit antidiabetic, anti-inflammatory, anticancer, antibacterial, and cholesterol-lowering properties [78], and antimicrobial activity against *S. aureus*, *B. subtilis*, and *K. pneumoniae*. One proposed mechanism is membrane disruption. Chromones, structurally similar to flavonoids but lacking the B-ring, are also known for their antitumor activity, potentially due to the carbonyl group in the pyrone ring [79].

## 4. Materials and Methods

### 4.1. Plant Collection and Extraction

The leaves of the selected plants (Table 1) were collected at the Lowveld National Botanical Garden, Mbombela, Mpumalanga Province, South Africa (Table 1). Plant material was authenticated by Dr. Bronwyn Egan at the Larry Leach Herbarium, University of Limpopo. The present study is primarily based on research originally conducted for a postgraduate thesis [91]. The fresh leaves were dried at room temperature, ground using a blender (LB-20, Labex Pty Ltd., Johannesburg, South Africa) stored in airtight containers away from light. The powdered leaf samples (1 g) were extracted with 10 mL of solvents spanning a polarity gradient: *n*-hexane, dichloromethane, acetone, methanol (all SupraSolv^®^, Darmstadt, Germany), and distilled water. Suspensions were agitated for 10 min at 200 rpm in a shaking incubator (Series 25, New Brunswick Scientific Co., Inc., Edison, NJ, USA). Extracts were filtered through Whatman No. 1 paper into pre-weighed glass vials and dried under a fan at ambient temperature. The dried residues were weighed to calculate yield and subsequently dissolved in acetone (SupraSolv^®^, Darmstadt, Germany) to obtain stock solutions at 10 mg/mL.

### 4.2. Quantification of Phytochemicals (Phenolic, Tannin and Flavonoid Content)

#### 4.2.1. Total Phenolic Content

Stock extracts (10 mg/mL) were first diluted to 5 mg/mL in acetone. From this dilution, 100 µL was transferred into a fresh test tube using a micropipette. To the mixture, 900 µL of distilled water and 100 µL of Folin–Ciocalteu reagent (Sigma-Aldrich^®^, St. Louis, MO, USA) were added. Calibration standards were prepared from gallic acid (Sigma-Aldrich^®^, St. Louis, MO, USA) over a concentration range of 0.078–1.25 mg/mL. Finally, 1 mL of 7% sodium carbonate solution (Sigma-Aldrich^®^, St. Louis, MO, USA) and the samples were incubated at room temperature in the dark for 30 min. The absorbance was read using the ultraviolet or visible (UV/VIS) spectrophotometer (Genesys 10S UV-VIS, Menlo Park, CA, USA) against the blank at 550 nm [92].

#### 4.2.2. Total Tannin Content

Total tannin contents of the selected plant extracts were determined using the Folin–Ciocalteu method. Plant extract (10 mg/mL) (50 μL) was added into a test tube with 3.8 mL of distilled water. The Folin–Ciocalteu reagent (Sigma Aldrich^®^, St. Louis, MO, USA) (250 µL) was added, and the mixture was vortexed. Then, 0.5 mL of 35% sodium carbonate (Na_2_CO_3_) solution (Supelco^®^, Bellefonte, PA, USA) was added. Distilled water was added to make the volume up to 10 mL. Gallic acid (Sigma Aldrich^®^, St. Louis, MO, USA) was used to prepare standard solutions (1.000–0.0625 mg/mL), which were also prepared in the same way. The samples were incubated at room temperature in the dark for 30 min. Absorbance of the samples against the blank were measured at 725 nm. The total tannin content was expressed mg GAE/g extract which was calculated using the formula obtained from the standard curve (y=1.4763x+0.0026, $R^{2}=0.9967$). The experiment was performed in triplicates and repeated three times [92]. Y is the dependent variable (absorbance), X is the independent variable (tannin content) and R is the coefficient.

#### 4.2.3. Total Flavonoid Content

The total flavonoid content of the plant extracts was determined using the aluminum chloride colorimetric assay. Exactly, 100 µL of 10 mg/mL plant extract was added to 4.9 mL of distilled water. Followed by 300 µL of 5% sodium nitrite (NaNO_2_) (Supelco^®^, Bellefonte, PA, USA) and incubated at room temperature for 5 min. Exactly 300 µL of 10% aluminum chloride (ALCl_3_) (Sigma Aldrich^®^, St. Louis, MO, USA) (dissolved in distilled water) was added to each reaction mixture and were incubated at room temperature for 5 min. Briefly, 2 mL of 1 M sodium hydroxide (NaOH) (Supelco^®^, Bellefonte, PA, USA) was added in each tube. The reaction mixtures were made to 10 mL using distilled water. Different concentrations (0.5–0.0313 mg/mL) of Quercetin (Sigma-Aldrich) were prepared as standard and absorbance readings of the samples were measured at 510 nm using the UV/VIS spectrophotometer (Thermo Scientific, CAT:840-209800, Waltham, MA, USA, Genesys 10S UV-VIS, Menlo Park, CA, USA) against the blank. The experiment was performed in triplicates, repeated three times, and total flavonoid content was expressed in milligram quercetin equivalence/gram of extract (mg QE/g extract) using the formula obtained from the standard curve (y=0.6371x+0.0049, $\;R^{2}=0.9972$) [92]. Y is the dependent variable (absorbance), X is the independent variable (flavonoid content) and R is the coefficient.

### 4.3. Quantitative Antioxidant Activity

#### 4.3.1. DPPH Free Radical Scavenging Activity Assay

The extracts were prepared in 1 mL acetone at concentrations (15.63–250 µg/mL). A volume of 2 mL of 2,2-diphenyl-1-picrylhydrazyl DPPH solution (0.2 mmol/L; Sigma-Aldrich^®^, St. Louis, MO, USA), was added to each sample. The mixtures were incubated at room temperature in the dark for 30 min. A control was prepared by replacing the extract with distilled water [93]. The absorbance of the solutions was recorded at 517 nm. L-ascorbic acid was included as the reference standard. Radical scavenging activity was expressed as percentage inhibition, calculated according to Equation (1):

% inhibition = [(Ac − As)/Ac] × 100(1)

where Ac is the absorbance of the control and As is the absorbance of the extract. The half-maximal effective concentration (EC_50_, µg/mL) was determined by plotting percentage inhibition against extract concentration. All experiments were performed in triplicate.

#### 4.3.2. Ferric Reducing Power (FRP) Assay

Plant extracts concentrations (39–625 µg/mL) were prepared. The procedure involved combining 2.5 mL of each concentration with 2.5 mL of 0.2 M sodium phosphate buffer (pH 6.6) and 2.5 mL of a 1% (*w*/*v*) potassium ferricyanide solution (Sigma Aldrich^®^, St. Louis, MO, USA). After being thoroughly vortexed, these mixtures were incubated at 50 °C for 20 min followed by the addition of 2 mL of a 10% (*w*/*v*) trichloroacetic acid solution (Sigma Aldrich^®^, St. Louis, MO, USA). Following the addition of the acid, the samples were centrifuged at 3000 rpm for 10 min. To supernatant (5 mL), 1 mL of a 0.1% (*w*/*v*) ferric chloride solution (Sigma Aldrich^®^, St. Louis, MO, USA) was added. The resulting solution was vortexed, and its absorbance was measured at 700 nm using a spectrophotometer [94]. For the blank solution, acetone was used instead of the plant extract. The experiment was conducted in triplicate.

### 4.4. Quantitative Antimycobacterial Activity Assay

#### 4.4.1. Broth Micro-Dilution Assay

The antimicrobial activity of plant extracts was evaluated using a serial broth microdilution assay [95] against *Mycobacterium smegmatis* (ATCC 1441). The bacterial strain was originally sourced from Professor Green at the University of Johannesburg, Department of Biotechnology and Food Technology. The microorganism was routinely maintained at 4 °C on Middlebrook 7H10 agar (Glentham Life Sciences Ltd, Corsham, UK) plates enriched with oleic albumin dextrose catalase (OADC) (Sigma Aldrich^®^, St. Louis, MO, USA). For the bioassays, a colony was cultured in Middlebrook 7H9 base broth (Glentham Life Sciences Ltd, Corsham, UK) supplemented with glycerol and OADC, and then incubated at 37 °C for 24 h. To establish a range of test concentrations, 100 µL of each 10 mg/mL plant extract was subjected to a 50% serial dilution with 100 µL of sterile distilled water in a 96-well plate, resulting in concentrations from 2.5 mg/mL down to 0.02 mg/mL. Following this, 100 µL of *M. smegmatis* culture (with an optical density of 0.8) was added to each well. Rifampicin and acetone were included as the positive and negative controls, respectively. The plates were incubated for 24 h at 37 °C. To visualize bacterial growth, 40 µL of a 0.2% p-iodonitrotetrazolium violet (INT) (Glentham Life Sciences Ltd, Corsham, UK) solution was introduced into each well. A final incubation of 30 min at 37 °C was performed, after which the plates were examined for clear wells, which indicated the complete inhibition of bacterial growth. This assay was performed in triplicate, and the MIC was recorded as the lowest concentration of a plant extract that successfully prevented the growth of *M. smegmatis*.

#### 4.4.2. The Antibacterial Interaction Effects

The antibacterial interaction effects of the *n*-hexane extracts were evaluated by preparing various combinations. Mixtures of these extracts, each at an initial concentration of 10 mg/mL, were combined in a 1:1 ratio. MIC values for these combinations were then determined to ascertain any synergistic or antagonistic interactions between the plants. The specific method for determining the MIC values was consistent with the procedure outlined in Section 4.4.1. The combined antimicrobial effect of the 1:1 plant combination was assessed by calculating the Fractional Inhibitory Concentration (FIC) Index. This index was determined by summing the individual FIC values for each component of the combination, denoted as FIC (i) and FIC (ii). The final FIC index was then used to classify the nature of the interaction. An index of ≤0.50 indicated a synergistic effect, while a value between 0.50 and 1.00 was considered additive. An index between > 1.00 and 4.00 was classified as indifferent, and a value > 4 signified an antagonistic interaction [96].

FIC (i) = MIC of (a) in combination with (b)/MIC of (a) independently(2)

FIC (ii) = MIC of (b) in combination with (a)/MIC of (b) independently(3)

### 4.5. Cell Viability Assay

The cell viability was assessed using the 3-(4,5-dimethylthiazol-2-yl)-2,5-diphenyltetrazolium bromide (MTT) assay [97]. The THP-1 monocytes (CelloNex, Johannesburg, South Africa) were first diluted to a concentration of 5 × 10^4^ cells/mL with RPMI(Merck, Modderfontein, South Africa) supplemented with 10% fetal bovine serum (FBS) (Merck, Modderfontein, South Africa). To induce their differentiation into adherent macrophage-like cells, the monocyte culture was adjusted to 2 × 10^5^ cells/mL and then pre-treated with 50 ng/µL of phorbol-12-myristate-13-acetate (PMA) (Sigma Aldrich^®^, St. Louis, MO, USA) and incubated at 37 °C with 5% CO_*2*_ 72 h. To increase macrophage marker expression, it is crucial to halt this differentiation process after 48 h of treatment [98]. After differentiation, the cells were washed with 1X phosphate-buffered saline (PBS) and fresh media was then added, followed by an additional 24 h incubation period [99]. Extract stock solutions (250 mg/mL) in dimethyl sulfoxide (DMSO) (Supelco^®^, Bellefonte, PA, USA) were diluted to 1 mg/mL using complete media, ensuring the final concentration of DMSO was maintained at 0.25%. A volume of 100 µL of the plant extracts (1000, 500, and 100 µg/mL) was then added to the wells containing the cell cultures. The plates were incubated for 24 h at 37 °C in 5% CO_*2*_. Following the incubation, the MTT assay was performed by adding 20 µL of a 0.5 mg/mL MTT (Merck, Modderfontein, South Africa) solution to each well, and the plates were incubated for 4 h. Subsequently, 100 µL of DMSO was added and left for one hour to dissolve the purple formazan crystals. The absence of this purple color or a clear appearance in the wells indicated the cytotoxic effect of the samples. The absorbance of the wells was then measured at 540 nm using a Promega microtiter plate reader, allowing for a comparison between the viability of treated and untreated cells.

### 4.6. Bioassay-Guided Fractionation Using Column Chromatography

#### 4.6.1. Column Chromatography Fractionation

Since *R. officinalis* exhibited strong antimycobacterial activity, it was selected for further analysis and isolation of its bioactive compounds. Serial exhaustive extraction was employed to extract bioactive constituents from 1 kg of ground *R. officinalis* leaf material. The plant material was first extracted with 6 L of *n*-hexane in an extraction bottle. The *n*-hexane extraction was repeated twice, with each extraction involving vigorous shaking for 3 h. The same plant residue was subsequently extracted using dichloromethane, followed by acetone and methanol. All extracts were concentrated using a rotary evaporator (Büchi R-114) (Marshall Scientific, Hampton, VA, USA), dried under a stream of cold air, reconstituted in acetone, and evaluated for antimycobacterial activity.

The combined dichloromethane extracts (D1, D2, and D3) were subjected to a first round of open-column chromatography [100]. A glass column (3 cm radius × 59.5 cm height) was packed with silica gel 60 (particle size 0.063–0.200 mm; Fluka) using 100% hexane. Elution of chemical constituents was performed with 1.6 L of solvent systems in the following sequence: *n*-hexane, *n*-hexane:ethyl acetate, ethyl acetate:methanol, and methanol. The collected fractions were concentrated using a rotary evaporator (Büchi R-114), and any remaining solvent was removed by drying under a stream of cold air. The antibacterial activity of the fractions was evaluated using a serial broth microdilution assay.

Significant antimycobacterial activity was observed in several fractions, with the most potent being Fraction 4 (70% *n*-hexane: 30% ethyl acetate) and Fraction 5 (50% n-hexane: 50% ethyl acetate), both of which had a MIC of 0.08 mg/mL. The fraction containing 30% *n*-hexane: 70% ethyl acetate had an MIC value of 0.3125 mg/mL. The bioactive compounds were identified in fractions 7 through 11. These specific fractions, which were eluted using mobile phases consisting of 10% *n*-hexane: ethyl acetate, 100% ethyl acetate, and 90–70% ethyl acetate: methanol, were found to exhibit both potent antibacterial activity and identical chemical profiles. Fractions 7–11 were combined (17.46 g) for further fractionation of bioactive compounds. The sub-fractions’ chemical profiles were determined using TLC plates, and fractions with the same chemical profiles were combined into groups 1–4 and tested for antibacterial activity.

#### 4.6.2. Preparative TLC

Groups 3 and 4 had the same chemical profile and antimycobacterial activity; they were selected and mixed, then separated using preparative TLC silica gel glass plates (Silica gel 60 F254) (Merck, Modderfontein, South Africa) in 80% *n*-hexane: 20% ethyl acetate mobile system. An ultraviolet light was used to visualize the compounds at 254 nm and 365 nm. The compounds were scraped off the plate and dissolved in ethyl acetate. The purity of the compounds was evaluated on TLC plates, and antimycobacterial activity validated using the procedure outlined in Section 4.4.1.

#### 4.6.3. Structural Elucidation of Isolated Compounds

Structural characterization of the isolated compounds was carried out at the Department of Chemistry, University of Limpopo. For analysis, each compound (4 mg) was resuspended in chloroform and analyzed on a Bruker 400 MHz NMR Spectrometer at a temperature of 295.5 K, using the solvent chloroform as a reference signal. The spectroscopic data collected (Figures S1 and S2) were subsequently sent to the Department of Chemical and Forensic Sciences at Botswana International University of Science and Technology to assist with the structural elucidation of the purified compounds (Figure S3). The comprehensive analysis included both 1-dimensional NMR (1H, 13C and DEPT 135) and 2-dimensional NMR (HMBC, HSQC, and COSY) experiments (Tables S1 and S2). ChemDraw (Version 20.1) was used to draw and predict the H and C resonances of proposed structures and chromones from the Literature, such as reference [89,90] to narrow down on the most probable structure.

### 4.7. Statistical Analysis

Statistical analysis was conducted using GraphPad Prism version 8.9. For one-way comparisons, a one-way analysis of variance (ANOVA) was performed, which was followed by Dunnett’s multiple comparison test. For other analyses, a two-way ANOVA was used, with a subsequent Tukey’s multiple comparison post hoc test where *p*-value < 0.05 was considered significant.

## 5. Conclusions

This study demonstrated that selected medicinal plants traditionally used in Limpopo Province possess significant antioxidant and antimycobacterial activities. Among the evaluated species, *R. tridentata* exhibited the highest total phenolic and tannin content in aqueous extracts. *R. officinalis* showed the highest flavonoid content in methanolic extracts. *X. caffra* displayed the strongest antioxidant activity, and *R. officinalis* demonstrated notable antimycobacterial activity across multiple extracts. Notably, the combination of *R. officinalis* and *Z. capense* exhibited an additive antimycobacterial effect. Bioassay-guided fractionation led to the isolation of antimycobacterial compounds; β-Sitosterol and 1-(4-oxo-2-vinyl-4H-chromen-7-yl)propan-2-yl palmitate from *R. officinalis* leaves. Cytotoxicity testing confirmed acceptable safety margins for most extracts, particularly the acetone extract of *R. tridentata*, which showed the highest percentage of cell viability. Overall, the findings affirm the pharmacological relevance of these species and highlight their potential as sources of lead compounds for developing safer, plant-based antimycobacterial agents. Further studies, including Liquid Chromatography–Mass Spectrometry analysis of the active extracts, ADME studies, and validation of the activity on *M. tuberculosis* (H37Rv) are recommended to advance these leads toward clinical relevance.

## Figures and Tables

**Table 1:** Plants used in the study.

Plant Name	Family Name	Common Names	Parts Used	Traditional Use	References
*Ziziphus mucronata* (UNIN 1220383)	Rhamnaceae	Buffalo thorn (Eng.); umphafa, (isiZulu); umphafa (isiXhosa); mokgalô, moonaona (N Sotho)	Leaves, roots, and bark	Body pains, cough, respiratory infections, and chest problems	[22]
*Zanthoxylum capense* (UNIN 1220384)	Rutaceae	small knobwood (Eng.); umnungamabele (isiZulu); monokwane (Sotho)	Leaves, roots, and bark	Cough	[24]
*Ximenia caffra* (UNIN 1220385)	Olacaceae	Sour plum (Eng.) umThunduluka-obmvu (Zulu); Morokologa (Northern Sotho)	Leaves and roots	Treat constipation, leprosy, and stomach pains	[31]
*Rosmarinus officinalis* (UNIN 1220072)	Lamiaceae	Rosemary	leaves	Chest pain, cough, and fever	[33,34]
*Rhoicissus tridentata* (UNIN 1220077)	Vitaceae	Wild grape (English), isinwazi (IsiZulu), and umnxeba (isiXhosa)	Tubers, leaves, and stem (wood)	Crushed, mixed with warm water and taken orally to treat TB-related symptoms	[33]

**Table 2:** Total phenolic, tannin and flavonoid content of selected plants.

Solvents	Total Phenolic mg GAE/g Extract	Total Tannin mg GAE/g Extract	Total Flavonoid (mg QE/g Extract
*Zanthoxylum capense*			
H	5.19 ± 1.57 ^a^	5.06 ± 0.81 ^a,b^	2.68 ± 0.57 ^a,b^
D	20.98 ± 3.72 ^a^	9.15 ± 1.51 ^a,b,c,d^	34.60 ± 2.18 ^g,h,i^
A	27.80 ± 2.97 ^a,b^	2.69 ± 1.41 ^a^	17.07 ± 10.41 ^c,d,e,f^
M	12.74 ± 0.67 ^a^	0.77 ± 0.18 ^a^	6.45 ± 1.10 ^a,b,c^
W	14.54 ± 1.58 ^a^	13.51 ± 0.34 ^a,b,c,d,e^	−4.69 ± 0.57 ^a^
*Rhoicissus tridentata*			
H	256.42 ± 15.18 ^h^	31.66 ± 1.22 ^f,g^	8.02 ± 1.40 ^b,c,d^
D	139.80 ± 15.59 ^e,f^	23.49 ± 4.26 ^d,e,f^	20.00 ± 2.12 ^d,e,f^
A	139.26 ± 17.07 ^e,f^	26.90 ± 2.91 ^e,f,g^	0.64 ± 0.16 ^a,b^
M	236.28 ± 20.57 ^h^	52.52 ± 8.67 ^h^	6.40 ± 1.00 ^a,b,c^
W	335.20 ± 8.26 ^i^	103.48 ± 7.36 ^i^	6.40 ± 3.44 ^a,b,c^
*Ziziphus mucronata*			
H	36.34 ± 6.92 ^a,b,c^	9.96 ± 1.22 ^a,b,c,d^	2.16 ± 1.27 ^a,b^
D	66.57 ± 4.96 ^c,d^	13.71 ± 2.37 ^a,b,c,d,e^	23.82 ± 1.41 ^f,g^
A	37.15 ± 2.96 ^a,b,c^	10.07 ± 0.22 ^a,b,c,d^	9.59 ± 1.81 ^b,c,d,e^
M	63.58 ± 8.22 ^b,c,d^	6.10 ± 0.45 ^a,b^	4.99 ± 0.50 ^a,b,c^
W	84.76 ± 7.68 ^d^	7.77 ± 3.72 ^a,b,c^	6.89 ± 0.42 ^a,b,c^
*Rosmarinus officinalis*			
H	180.80 ± 11.91 ^g^	50.06 ± 6.99 ^h^	35.33 ± 0.42 ^g,h,i^
D	126.67 ± 2.26 ^e^	20.05 ± 6.37 ^b,c,d,e,f^	20.68 ± 4.99 ^e,f^
A	149.43 ± 3.19 ^e.f,g^	34.37 ± 10.10 ^f,g^	35.91 ± 4.18 ^g,h,i^
M	177.71 ± 30.34 ^f,g^	22.83 ± 12.50 ^c,d,e,f^	45.90 ± 11.04 ^j^
W	32.71 ± 1.14 ^a,b,c^	10.10 ± 1.51 ^a,b,c,d^	4.69 ± 0.27 ^a,b^
*Ximenia caffra*			
H	133.78 ± 7.89 ^e^	9.53 ± 0.58 ^a,b,c,d^	19.90 ± 2.36 ^d,e,f^
D	147.01 ± 13.94 ^e,f,g^	3.53 ± 0.41 ^a^	7.45 ± 2.22 ^b,c^
A	163.27 ± 9.69 ^e,f,g^	10.17 ± 1.66 ^a,b,c,d^	35.28 ± 4.40 ^g,h,i^
M	229.76 ± 27.82 ^h^	11.50 ± 1.74 ^a,b,c,d^	37.16 ± 3.77 ^h,j^
W	225.62 ± 4.01 ^h^	41.48 ± 6.85 ^g,h^	28.79 ± 3.87 ^f,g,h^

GAE: milligram of gallic acid equivalence/gram of extract, QE: milligram of quercetin equivalence/gram of extract, H: hexane; D: dichloromethane; A: acetone; M: methanol; W: water. Values expressed as the mean ± standard deviation (SD) of triplicate experiments; values with different letter superscripts in a column are significantly different at *p* < 0.05; same letter superscript values in a column are not significantly different (*p* > 0.05).

**Table 3:** Antioxidant activity of the plant extracts.

Plant Species	Solvent	DPPH Free Radical Scavenging Activity		Ferric Reducing Antioxidant Power Activity	
		EC_50_ (µg/mL)	R^2^	EC_50_ (µg/mL)	R^2^
*Z. mucronata*	H	28.08	0.98	299.5	0.95
	D	8.709	0.97	297.3	0.97
	A	79.96	0.97	305.5	0.94
	M	33.80	0.98	309.8	0.97
	W	20.47	0.97	332.9	0.98
*X. caffra*	H	0.0094	0.98	2.490	0.94
	D	0.0039	0.94	2.164	0.99
	A	151.3	0.96	0.481	0.96
	M	10.19	0.96	2.442	0.93
	W	11.78	0.97	2.484	0.95
*R. officinalis*	H	0.0063	0.96	310.5	0.96
	D	8.601	0.97	274.9	0.97
	A	4.979	0.99	272.9	0.97
	M	0.0073	0.96	309.8	0.94
	W	197.0	0.98	353.4	0.99
*R. tridentata*	H	0.0058	0.93	238.4	0.87
	D	1014	0.97	297.5	0.97
	A	29.54	0.94	286.7	0.97
	M	33.98	0.99	301.6	0.97
	W	19.88	0.99	144.5	0.97
*Z. capense*	H	137.4	0.92	76.37	0.87
	D	209.2	0.99	212.0	0.96
	A	-	-	288.5	0.98
	M	-	-	310.9	0.98
	W	-	-	326.3	0.89
Ascorbic acid		0.0038	0.96	3.042	0.93

H: hexane, D: dichloromethane, A: acetone, M: methanol, W: water, EC_50_: half maximal effective concentration, R^2^: coefficient of determination, (-): not determined.

**Table 4:** The MIC values of selected plant extracts in mg/mL against *M. smegmatis*.

	Plant Species					
Solvent	*Z. mucronata*	*R. officinalis*	*R. tridentata*	*X. caffra*	*Z. capense*	Rifampicin
Hexane	>2.5	0.08	0.16	0.31	>2.5	0.08
Dichloromethane	>2.5	0.08	0.31	>2.5	>2.5	
Acetone	1.25	0.08	>2.5	>2.5	>2.5	
Methanol	1.25	0.08	>2.5	>2.5	>2.5	
Water	>2.5	>2.5	>2.5	>2.5	>2.5	
Average	2.0	0.56	1.59	2.06	2.5	

**Table 5:** The FIC of selected plants using hexane extracts.

Combination	MIC (mg/mL)	FIC (i)	FIC (ii)	FIC Index	Outcome
RO:RT	0.63	7.81	4.01	11.82	Antagonistic
RO:ZM	0.63	7.81	0.25	8.06	Antagonistic
RO:XC	0.63	7.81	0.20	9.81	Antagonistic
RO:ZC	0.63	7.81	0.25	8.06	Antagonistic
RT:ZM	2.50	16.03	1.00	17.03	Antagonistic
RT:XC	2.50	16.03	8.00	24.03	Antagonistic
RT:ZC	2.50	16.03	1.00	17.03	Antagonistic
ZM:XC	2.50	1.00	8.00	9.00	Antagonistic
ZM:ZC	2.50	1.00	1.00	2.00	Indifferent
XC:ZC	2.50	8.00	1.00	9.00	Antagonistic

*R. officinalis* (RO), *R. tridentata* (RT), *X. caffra* (XC), *Z. mucronata* (ZM), *Z. capense* (ZC), Fractional Inhibitory Concentration (FIC), and Minimum Inhibitory Concentration (MIC).

**Table 6:** MIC values obtained from serial exhaustive extraction of crude extracts and isolated compounds against *Mycobacterium smegmatis*.

Extract	MIC Value (mg/mL)
n-Hexane 1 (H1)	0.182
n-Hexane 2 (H2)	0.156
n-Hexane 3 (H3)	0.156
Dichloromethane 1 (D1)	0.3125
Dichloromethane 2 (D2)	0.3125
Dichloromethane 3 (D3)	0.3125
Acetone 1 (A1)	1.125
Acetone 2 (A2)	2.5
Acetone 3 (A3)	2.5
Methanol 1 (M1)	1.875
Methanol 2 (M2)	2.5
Methanol 3 (M3)	2.5
Negative control (Acetone)	>2.5
Positive control (Rifampicin)	0.08
Isolated compounds	
Compound **1**	0.125
Compound **2**	0.25
Rifampicin	0.002
Acetone	>0.25

## Data Availability

The original contributions presented in this study are included in the article.

## Supplementary Materials

The following supporting information can be downloaded at: https://www.mdpi.com/article/10.3390/antibiotics14100965/s1, Figure S1: NMR spectra of isolated compound **1**. (**a**) 1H NMR spectrum, (**b**) 13C NMR spectrum, (**c**) DEPT 135 NMR spectrum, (**d**) COSY NMR spectrum, (**e**) HSQC NMR spectrum, (**f**) HMBC NMR spectrum; Figure S2: NMR spectra of isolated compound **2**. (**a**) ^1^H NMR spectrum, (**b**) ^13^C NMR spectrum, (**c**) COSY NMR spectrum; Figure S3: The phytochemical analysis of isolated compounds developed in 80% n-hexane: 20% ethyl acetate, sprayed with the vanillin-sulphuric acid reagent (Compound **1**) and visualised 365 nm (Compound **2**); Table S1: The summary of ^1^H and ^13^C spectroscopic data; Table S2: The summary of ^1^H and ^13^C spectroscopic data.

## Abbreviations

The following abbreviations are used in this manuscript:

ROS	Reactive oxygen species
XDR	Extensively drug-resistant
MDR	Multidrug resistant
NMR	Nuclear magnetic resonance
WHO	World Health Organization
TLC	Thin-layer chromatography
