# Supplementary material for: Isolation and In Vitro Pharmacological Evaluation of Phytochemicals from Medicinal Plants Traditionally Used for Respiratory Infections in Limpopo Province
Source: Antibiotics (Basel). 2025 Sep 25;14(10):965. doi: 10.3390/antibiotics14100965 (PMC12561057; doi:10.3390/antibiotics14100965)
Supplement: Supplementary file 1 [file antibiotics-14-00965-s001.zip › Figure S1.pdf]

a

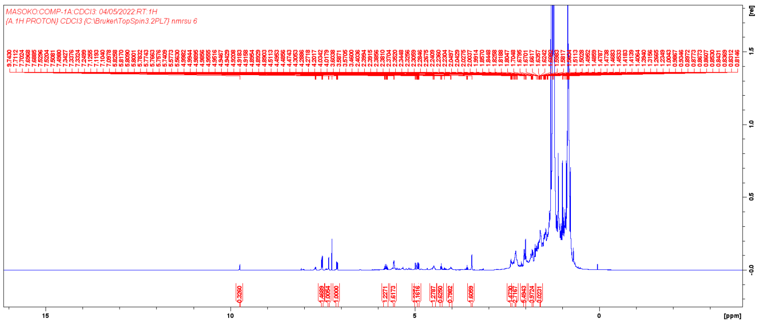
MASOKO:COMP-1A:CDCl3: 04/05/2022:RT:1H

b

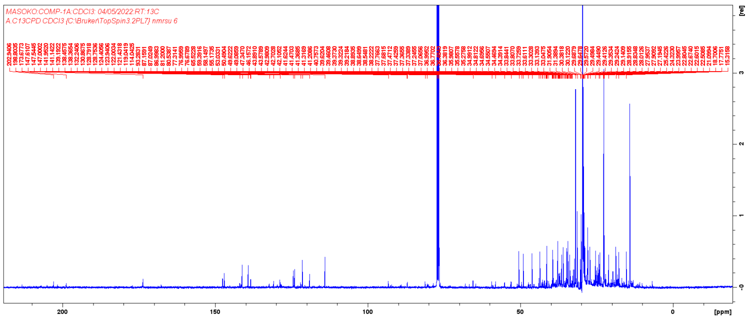
MASOKO:COMP-1A:CDCl3: 04/05/2022:RT:13C

c

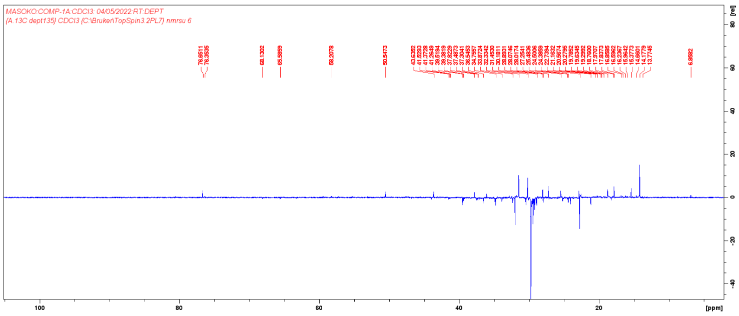
MASOKO:COMP-1A:CDCl3: 04/05/2022:RT:DEPT

d

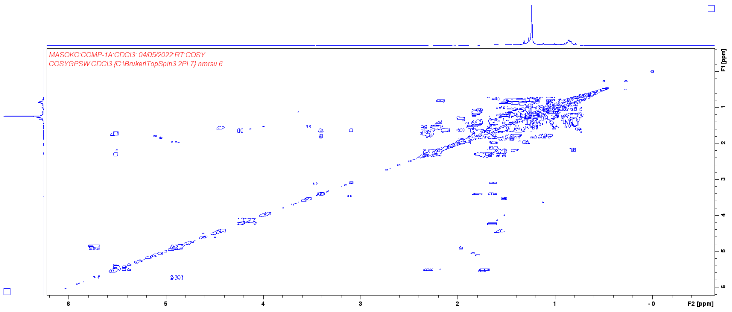
MASOKO:COMP-1A:CDCl3: 04/05/2022:RT:COSY

e

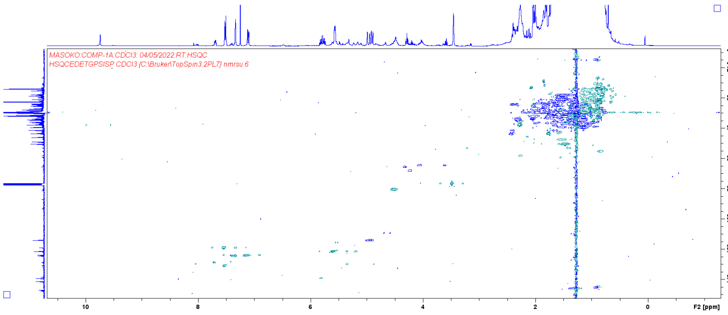
MASOKO:COMP-1A:CDCl3: 04/05/2022:RT:HSQC

f

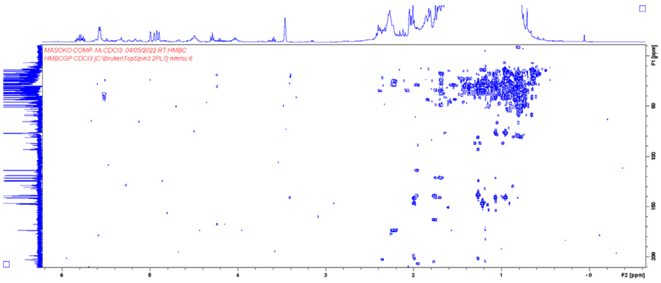

Figure S1. NMR spectra of isolated compound 1. (a) ^{1}H NMR spectrum, (b) ^{13}C NMR spectrum, (c) DEPT 135 NMR spectrum, (d) COSY NMR spectrum, (e) HSQC NMR spectrum, (f) HMBC NMR spectrum.
