# Supplementary material for: Isolation and In Vitro Pharmacological Evaluation of Phytochemicals from Medicinal Plants Traditionally Used for Respiratory Infections in Limpopo Province
Source: Antibiotics (Basel). 2025 Sep 25;14(10):965. doi: 10.3390/antibiotics14100965 (PMC12561057; doi:10.3390/antibiotics14100965)
Supplement: Supplementary file 1 [file antibiotics-14-00965-s001.zip › Figure S2.pdf]

a

b

c

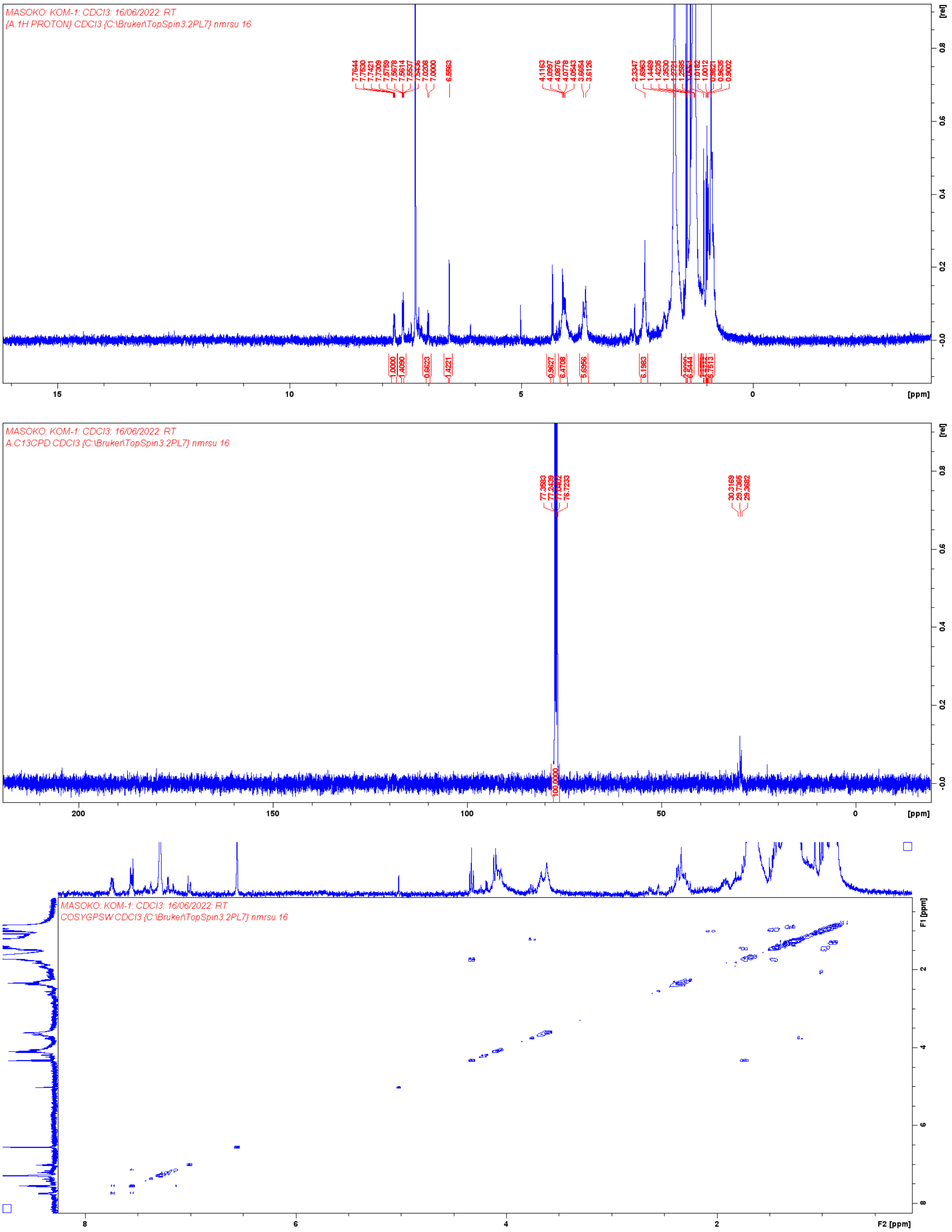

Figure S2. NMR spectra of isolated compound 2. (a) ^{1}H NMR spectrum, (b) ^{13}C NMR spectrum, (c) COSY NMR spectrum.
