# Supplementary material for: Isolation and In Vitro Pharmacological Evaluation of Phytochemicals from Medicinal Plants Traditionally Used for Respiratory Infections in Limpopo Province
Source: Antibiotics (Basel). 2025 Sep 25;14(10):965. doi: 10.3390/antibiotics14100965 (PMC12561057; doi:10.3390/antibiotics14100965)
Supplement: Supplementary file 1 [file antibiotics-14-00965-s001.zip › Figure S3.pdf]

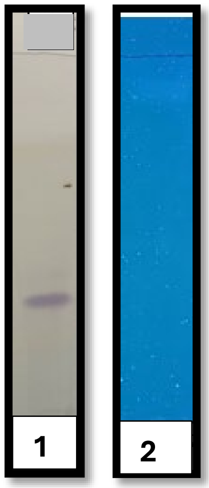

Figure S3. The phytochemical analysis of isolated compounds developed in 80% n-hexane: 20% ethyl acetate, sprayed with the vanillin-sulphuric acid reagent (Compound 1) and visualised 365 nm (Compound 2).
