# Supplementary material for: Isolation and In Vitro Pharmacological Evaluation of Phytochemicals from Medicinal Plants Traditionally Used for Respiratory Infections in Limpopo Province
Source: Antibiotics (Basel). 2025 Sep 25;14(10):965. doi: 10.3390/antibiotics14100965 (PMC12561057; doi:10.3390/antibiotics14100965)
Supplement: Supplementary file 1 [file antibiotics-14-00965-s001.zip › Table S1.pdf]

Table S1: The summary of ^{1}H and ^{13}C spectroscopic data.

Positions	Type	Chemical shift, δ (ppm) value			
		Ododo *et al.*, 2016		Compound 1	
		^{13}C	^{1}H	^{13}C	^{1}H
1	CH2	37.2	1.46 (m)	38.77	1.65
2	CH2	31.69	1.56 (m)	30.5	1.85
3	CH(OH)	71.82	3.54 (m)	79.01	3.21
4	CH2	42.33	2.32 (m)	41.92	2.32
5	C	140.77	-	137.87	-
6	CH	121.73	5.37 (overlapping, t)	125.79	5.23, d, J=3.5 Hz
7	CH2	31.93	2.04 (m)	31.91	2.15 (m)
8	CH	31.93	1.69 (m)	31.91	2.12 (m)
9	CH	50.16	1.55 (m)	47.86	1.91
10	C	36.51	-	36.66	-
11	CH2	21.11	1.52 (m)	21.16	1.65
12	CH2	39.80	1.51 (m)	39.41	1.65
13	C	42.34	-	41.57	-
14	CH	56.79	1.50 (m)	55.13	1.65(m)
15	CH2	24.33	1.58 (m)	23.55	1.74 (m)
16	CH2	28.27	1.85 (m)	27.94	1.99 (m)
17	CH	56.08	1.45 (m)	52.87	1.65 (m)
18	CH3	11.89	0.70 (s)	15.29	0.91 (s)
19	CH3	19.42	1.03 (s)	15.44	1.12 (s)
20	CH	36.17	1.60 (m)	36.94	1.74
21	CH3	18.84	0.94 (overlapping, d)	15.57	1.00
22	CH2	33.98	0.93 (m)	32.88	0.89 (m)
23	CH2	26.11	1.15 (m)	27.15	1.52
24	CH	45.86	1.38 (m)	47.40	1.59 (m)

25	CH	29.19	157 (m)	29.35	1.74
26	CH3	19.84	0.84 (overlapping, d)	18.24	0.91 (s)
27	CH3	19.06	0.86 (d)	17.00	0.97 (s)
28	CH2	23.10	1.10 (m)	22.68	1.52
29	CH3	12.01	0.82 (overlapping, t)	14.12	2.86
-	OH	-	1.98 (s)	-	1.98 (s)
