# Supplementary material for: Isolation and In Vitro Pharmacological Evaluation of Phytochemicals from Medicinal Plants Traditionally Used for Respiratory Infections in Limpopo Province
Source: Antibiotics (Basel). 2025 Sep 25;14(10):965. doi: 10.3390/antibiotics14100965 (PMC12561057; doi:10.3390/antibiotics14100965)
Supplement: Supplementary file 1 [file antibiotics-14-00965-s001.zip › Table S2.pdf]

Table S2: The summary of ^{1}H and ^{13}C spectroscopic data.

Positions	δ_H	δ_C
1	-	-
2	-	167.0
3	5.57, *d*, *J*= 5.7 Hz	121.4
4	-	173.0
4a	-	122.0
5	7.74, *d*, *J*= 8.4 Hz	119.0
6	7.12, *dd*, *J*= 2.5, 8.6 Hz	123.9
7	-	141.1
8	7.33, *t*, *J*= 2.0 Hz	124.4
8a	-	147.6
1′	4.00-4.24, *m*	44.0
2′	3.46, *m*	76.4
3′	1.23, *s*	25.4
1′′	4.93, *m*	138.3
2′′	a) 4.91, *dd*, *J*= 1.0, 10.2 Hz<br>b) 4.99, *dd*, *J*= 2.7, 17.1 Hz	114.0
